# Oncogenic human papillomaviruses

**DOI:** 10.1098/rstb.2016.0273

**Published:** 2017-09-11

**Authors:** Alison A. McBride

**Affiliations:** Laboratory of Viral Diseases, National Institute of Allergy and Infectious Diseases, National Institutes of Health, Bethesda, MD 20892, USA

**Keywords:** HPV, papillomavirus, cancer, keratinocyte

## Abstract

Human papillomaviruses (HPVs) are an ancient group of viruses with small, double-stranded DNA circular genomes. They are species-specific and have a strict tropism for mucosal and cutaneous stratified squamous epithelial surfaces of the host. A subset of these viruses has been demonstrated to be the causative agent of several human cancers. Here, we review the biology, natural history, evolution and cancer association of the oncogenic HPVs.

This article is part of the themed issue ‘Human oncogenic viruses’.

## 1. Viral life cycle

### (a) Human papillomavirus genome organization

**Figure 1.**
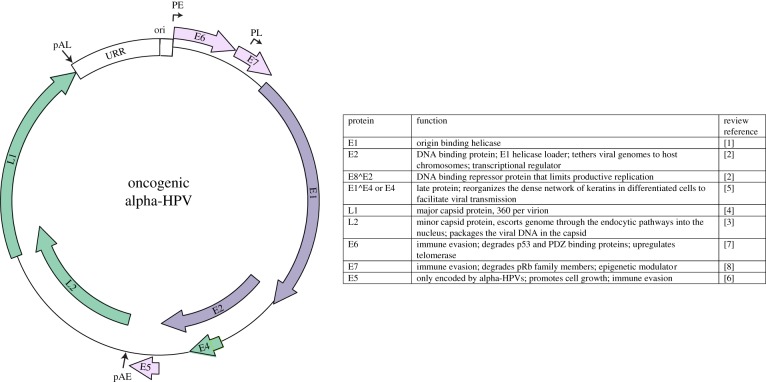
Viral genome. Map of an alpha-HPV genome. The URR (upstream regulatory region) contains the replication origin (ori). The early promoter (PE), late promoter (PL) and early and late polyadenylation sites (pAE and pAL) are indicated.

The family *Papillomaviridae* is a group of small, non-enveloped viruses with double-stranded DNA circular genomes that are mostly 7–8 kbp. All papillomaviruses encode four conserved core proteins: E1 and E2 are replication factors [1,2]; and L1 and L2 are capsid proteins [3,4]. In addition, the oncogenic HPVs encode accessory proteins: E4, E5, E6 and E7 [5–9]. These proteins modulate the cellular environment to make it more conducive for viral replication, and are important for immune evasion. The genome can be divided into three regions: the upstream regulatory region (URR) contains cis elements that control transcription and replication; the early region encodes the E1, E2, E4, E5, E6 and E7 proteins; and the late region encodes the L1 and L2 structural proteins. A typical alpha-HPV genome is shown in figure 1. The small genome is densely packed with overlapping open reading frames and *cis* regulatory elements. Transcription occurs in three waves, which are dependent on the differentiation status of the host cell [10]. Early transcription is initiated from early promoters situated just upstream from the early coding region and terminated at the early polyadenylation site. Intermediate transcription originates from the late promoter, and transcribes high levels of the E1 and E2 replication proteins, but still terminates at the early promoter. Late transcription uses both the late promoter and late polyadenylation site, and results in high-level expression of the L1 and L2 proteins.

### (b) Overview of human papillomavirus infectious cycle

**Figure 2.**
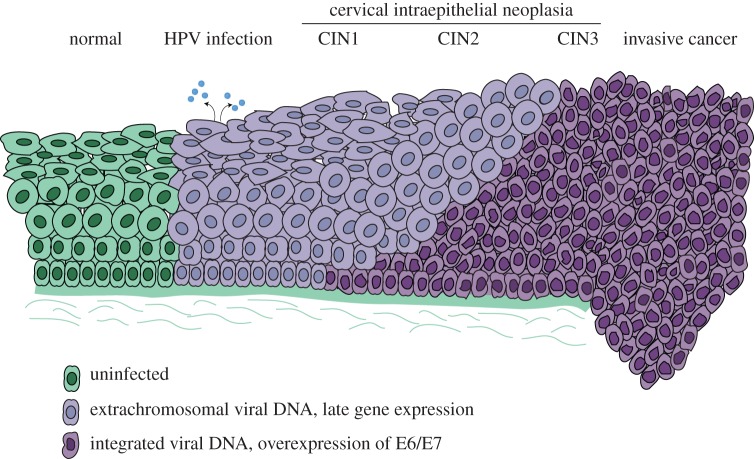
Oncogenic progression of cervical infection. Diagram of steps in progression from HPV infection to invasive cervical cancer.

HPVs infect cutaneous and mucosal sites and take advantage of the highly organized process of tissue renewal in stratified squamous epithelia. The virus infects the cells in the lower, basal layer of the epithelium through a micro-abrasion [11] and establishes a long-term, persistent infection within these cells. When these infected cells differentiate and move up towards the surface of the epithelium, high-level viral replication and gene expression is induced. Virions are assembled in the superficial layers and are released from the epithelium in viral-laden squames (figure 2). This strategy of infecting self-renewing cells ensures long-term viral persistence, while restricting high levels of viral proteins to more differentiated layers of the lesion is thought to help the virus escape detection by the immune system.

The HPV virion is an icosahedral capsid assembled from 360 molecules of the L1 protein [4]. The viral genome is packaged into the L1 capsid as a mini-chromosome assembled with host histones [12], and up to 72 copies of the L2 minor capsid protein [13]. HPVs are thought to access the basal cells through a fissure in the epithelium that exposes the basement membrane [11]. This tactic allows access to self-renewing cells and promotes cellular proliferation as part of the wound-healing process, which could aid in the establishment of the viral infection. The viral capsid initially interacts with heparin sulfate proteoglycans on the basement membrane, which induces a conformational change that allows the virus to bind to a (as yet unidentified) secondary receptor on the surface of the basal keratinocytes [14].

The virion is taken into the cell by endocytosis and is trafficked through the endosomal pathways to the trans-Golgi network. The virus is uncoated during trafficking and only the viral mini-chromosome, in complex with L2, enters the nucleus encased in a membrane vesicle [15]. The cell must undergo mitosis and nuclear envelope breakdown to allow the L2-genome complex to access the nucleus [16,17]. Similar to many other viruses, the L2-genome complex is next observed adjacent to ND10 nuclear bodies [18]. These bodies are thought to be important for intrinsic immune defence, but somewhat counterintuitively, they also are attractive locations for viruses to establish their replication and transcription programme [19]. Viruses often reorganize the components of the ND10 bodies, and likewise the HPV L2 protein displaces the Sp100 protein, and recruits Daxx [20], to induce a local environment suitable for initiation of viral transcription and replication.

Early viral transcripts encode the E1 and E2 replication proteins to support limited viral DNA amplification [21]. There are three phases of replication in the viral life cycle: first there is limited DNA amplification when the virus first infects the cell; next there is maintenance replication, when the viral genome replicates at a constant copy number in the proliferating cells of a lesion; and finally there is differentiation-dependent amplification when the viral DNA is replicated to high copy number to generate progeny virions [22]. E2 recruits and loads the E1 helicase onto the viral replication origin, but otherwise the virus relies on cellular proteins to synthesize viral DNA [22]. Both early and late amplification of viral DNA engages the cellular DNA damage response (DDR) [23,24] to support DNA synthesis. During the maintenance phase of replication, the E2 protein ensures that the low copy number viral genomes are efficiently partitioned to the daughter cells by tethering them to host chromatin [22]. To achieve this, E2 contains both a DNA binding domain that interacts with conserved sites (E2BS) in the viral genome, and a ‘transactivation’ domain that interacts with host chromatin [2]. Notably, this tethering mechanism is shared by other oncogenic viruses with extrachromosomal genomes, and is critical for persistent infection [22]. E2 is also the primary transcriptional regulator of the virus [2]. There are four highly conserved E2BS in the URR regulatory region of the oncogenic alpha-HPVs that are required for both replication and transcription functions of E2. E2 either activates or, more often, represses viral transcription [2]. All HPVs also encode an E2-derived protein E8^E2 that represses viral transcription and replication, thus restricting the viral life cycle and maintaining a low-level persistent infection [2].

E6 and E7 are the oncoproteins of the high-risk alpha-HPVs. These proteins are less well conserved, and more specialized than the core proteins of the virus. Through a multitude of interactions with cellular proteins, they both promote cellular proliferation and inactivate cell-cycle checkpoints to promote viral replication in differentiated cells [7,8]. E7 causes replication stress and epigenetically reprogrammes cellular circuits that result in oncogene-induced senescence [25], and there is evidence that viral-mediated inactivation of the pRb pathway is primarily to counteract this response [26]. E6 and E7 also disrupt interferon and NFkB signalling pathways, allowing the virus to persist and escape detection [27]. There is substantial overlap between immune signalling and tumour suppression pathways, leading to a hypothesis that persistent oncogenic viruses target these pathways primarily to escape immune detection, with the unfortunate side effect of oncogenic promotion [28]. The E5 protein can facilitate immune evasion by downregulating surface expression of proteins involved in antigen presentation, and can also promote proliferation by enhancing EGFR signalling pathways [6].

In the upper layers of an infected lesion, viral DNA is amplified to a high copy number. This phase requires induction of the DDR, which is thought to recruit repair factors that the virus can hijack to replicate its DNA [23,29]. The E4 protein is also expressed abundantly in the upper layers of the lesion, where it reorganizes the network of keratin filaments to facilitate virus release and transmission [5]. High levels of L1 and L2 result in the self-assembly of capsids that encapsidate viral DNA. Superficial cells containing arrays of virions are naturally sloughed from the surface of the epithelium.

## 2. Evolution of human papillomaviruses

### (a) Diversity and rate of evolution

**Figure 3.**
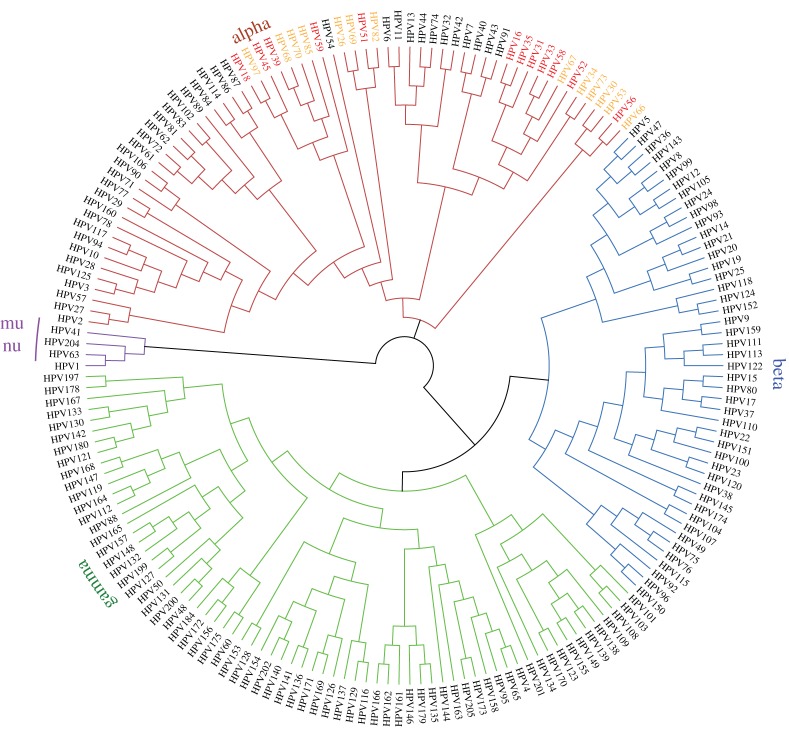
Phylogenetic tree based on the L1 nucleotide sequence of HPVs. Group 1 oncogenic alpha-HPVs are shown in red, and Group 2 in orange.

To date, the family *Papillomaviridae* contains 49 genera and over 300 individual human and animal papillomavirus types (http://pave.niaid.nih.gov/). Over 200 types are HPVs, which are organized into five phylogenetic genera named alpha, beta, gamma, mu and nu, shown in figure 3 [30]. Papillomaviruses evolve extremely slowly with a mutation rate only five times that of the host species [31]; this rate is balanced by the relatively rapid generation times of papillomaviruses versus the highly constrained nature of the viral genome. Moreover, while HPVs use host DNA polymerases to replicate their genomes, this might entail polymerases specialized for DNA repair [29].

### (b) Host range and niche adaptation

Until recently, it was thought that papillomavirus infection was restricted to Amniotes (mammals, birds and reptiles), indicating that virus and host had been coevolving for over 300 Myr [32]. However, the recent isolation of a fish (Actinopterygii) papillomavirus extends this timeline back for an additional 120 Myr [33]. The five genera of human papillomaviruses are dispersed through the phylogenetic tree, which indicates that these lineages diverged prior to speciation of the host *Homo sapiens* [32]. Current thinking is that papillomaviruses originally diverged to take advantage of emerging ecological niches in the wide-ranging epidermises and epidermal appendages of vertebrates. Subsequently, viruses within each genus continued to coevolve and adapt to a specific niche of their host [31,32]. It is this final cospeciation and niche adaptation, often tropic to vulnerable cells, which likely gave rise to the oncogenic HPVs.

### (c) Taxonomy of oncogenic human papillomaviruses

**Figure 4.**
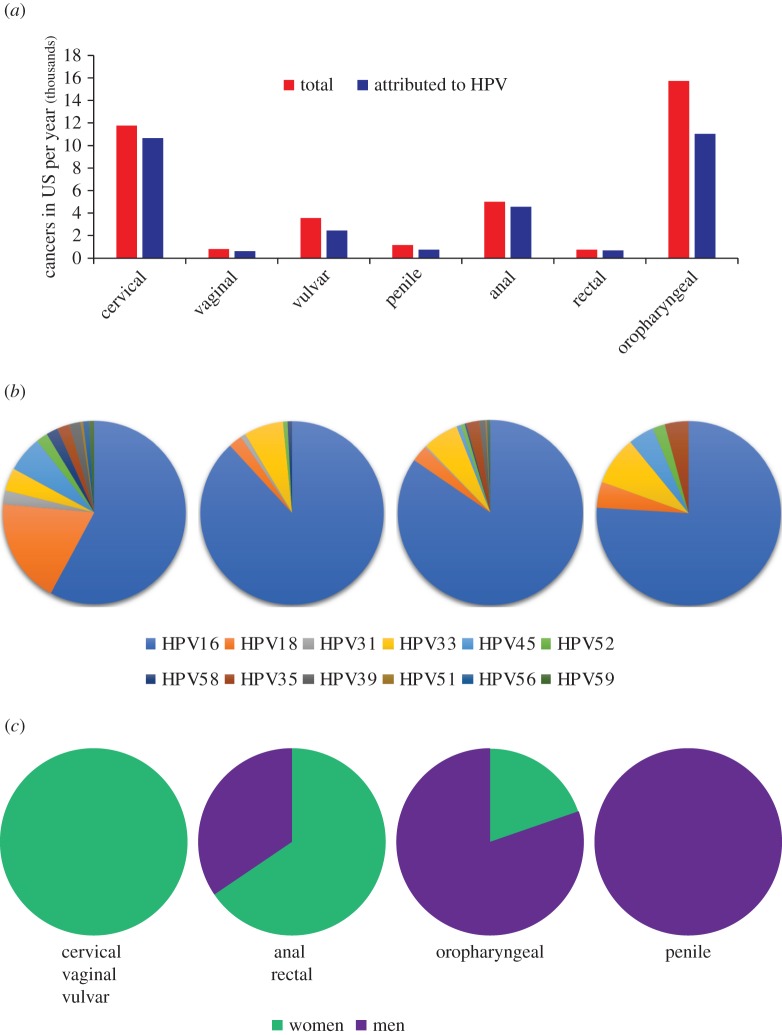
Cancer statistics. (*a*) HPV-associated cancer incidence in the US 2008–2012 (data from Viens *et al*. [34]). (*b*) Distribution of oncogenic HPV types in different cancers (data from Saraiya *et al*. [35]). (*c*) Relative proportion of HPV-associated cancer cases based on gender (data from Viens *et al*. [34]).

The host specificity and benign nature of most papillomavirus infections is indicative of a long virus–host association [32]. Although the majority of animal papillomaviruses have been isolated from clinically apparent lesions, the much greater sampling depth of human epithelia reveals that most papillomaviruses give rise to subclinical or asymptomatic infections. Out of the five genera of HPVs, four (beta, gamma, mu and nu) contain only viruses that infect cutaneous epithelia. The fifth, the alpha genus, is unique in that it contains HPVs tropic to both cutaneous and mucosal epithelia. The oncogenic HPVs are a subset of the mucosotropic alpha-HPVs. The 12 ‘high-risk’ oncogenic HPVs are shown in red in figure 3, and the possibly/probably oncogenic HPVs are shown in orange. Figure 4*a* shows the different human cancers associated with HPV infection, and figure 4*b* shows the distribution of oncogenic HPV types found associated with different cancer sites [35].

### (d) Variant human papillomaviruses

Individual HPV types have less than 90% nucleotide sequence identity in the L1 gene compared with any other named HPV type [36]. However, variant lineages have between 1 and 10% differences in the L1 region [37]. Remarkably, although HPV evolution is very slow, genetic drift of HPVs can be used to monitor the migration of ancient human populations, and indicates that variant diversity within an HPV type has been evolving for over 200 000 years [38]. Many studies have attempted to identify oncogenic HPV variants with the highest propensity to cause cancer; but viral-mediated oncogenesis is a multifaceted process with contributions from viral oncogene properties, viral persistence and host immunity. It is crucial to have a consistent method to number genomes, and classify variants [37], so that the huge datasets obtained from next-generation sequencing techniques and epidemiological data can be reliably compared and used to identify subtle correlations that might provide important insight into the multifactorial process of viral-mediated oncogenesis.

## 3. Human papillomavirus-associated cancers

### (a) Alpha human papillomaviruses

Over 15% of human cancers can be attributed to infectious agents, and almost one-third of these are due to infection by HPVs [39]. HPVs are highly associated with the development of cervical cancer, as well as vaginal, vulvar, anal, rectal, penile and oropharyngeal cancers [34,40]. In the 2012 the International Agency for Research on Cancer (IARC) Monographs on the Evaluation of Carcinogenic Risks to Humans, 12 HPVs were declared carcinogenic (Group 1), and an additional 13 were classified as either probably, or possibly, carcinogenic (Group 2A and B) based on limited evidence and/or their close phylogenetic placement with other carcinogenic HPVs [40]. The oncogenic viruses in each category are shown on the phylogenetic tree in figure 3.

### (b) Beta human papillomaviruses

There has long been debate as to whether HPV types from the beta genus are associated with skin keratinocyte carcinomas (KC), in particular squamous cell carcinoma [41,42]. IARC have declared that, at least in individuals with the genetic disease epidermodysplasia verruciformis (EV), two beta-HPV types are possibly carcinogenic (Group 2). But so far there is insufficient evidence for these viruses to be classified as carcinogenic in normal individuals [40]. It has been very difficult to determine whether beta-HPVs are the etiological agent of KCs because, unlike the oncogenic alpha-HPVs, they are not required for maintenance of tumours [41]. Furthermore, they are not found integrated in tumour cells, and no predominant HPV type is consistently found associated with KC. Nevertheless, there is some evidence that beta-HPV could play a role in the initiation of carcinogenesis [41].

### (c) How do human papillomaviruses cause cancer?

Although many processes ultimately affect persistent HPV infection, the E6 and E7 proteins are necessary and sufficient for HPV-mediated oncogenesis. All HPV E6 and E7 proteins bind to a plethora of cellular proteins and teasing apart the precise interactions that make an E6 or E7 protein oncogenic has not been simple [43–46]. All papillomaviruses drive cellular proliferation in the upper layers of an epithelium to promote viral DNA amplification; however, the oncogenic HPVs also promote cell-cycle entry, and inactivate cell-cycle checkpoints, in the lower layers of an infected epithelium [45]. The resulting genetic instability in these proliferating cells has much more serious consequences compared to the upper terminally differentiated cells. Key functions of the HPV oncogenes are immune evasion, E7-mediated degradation of pRB family members, E6-mediated degradation of p53 and PDZ binding domain proteins and E6-mediated upregulation of telomerase [43–46] and these are listed in table 1. By contrast, the E6 and E7 proteins of the beta-HPVs act as cofactors by inhibiting the cell-cycle arrest and repair of UV-induced DNA damage [53], but they are not required for maintenance of the tumour phenotype.

**Table 1.** Major properties of the HPV E6 and E7 oncogenes.

oncogene	property	reference
**E7**	epigenetic reprogramming of cells by upregulation of KDM6A and KDM6B	[25]
	abrogation of pRb/E2F pathway by pRb degradation	[47]
	induction of DDR in differentiated cells to promote viral DNA amplification	[48]
	inhibition of innate immune response	reviewed in [49]
**E6**	proteasome-mediated degradation of p53	[50]
	induction of telomerase expression	[51]
	degradation of PDZ domain proteins involved in cell polarity	reviewed in [52]
	inhibition of innate immune response	reviewed in [49]

## 4. Natural history of human papillomavirus infection and cancer progression

### (a) Initial and persistent infection of the cervix

The oncogenic alpha-HPVs are sexually transmitted and about 30% of young women become infected within 24 months of their first sexual exposure [54]. Infection can result in mild cytological cervical abnormalities but about 90% will clear within 2 years [54]. However, long-term persistent infection, beyond this time period, places individuals at high risk for cervical intraepithelial neoplasia (CIN). Figure 2 depicts oncogenic progression from infection through CIN stages 1–3, and finally to invasive cancer. It is estimated that about one-third of CIN3 lesions will progress to invasive cancer within 10–20 years [54].

### (b) Immune detection and clearance

During the period of persistent infection, there is no apparent immune detection of the virus [55]. This is in part, due to the viral life cycle itself, which ensures that high levels of viral activity occur only in the upper, differentiated cell layers that are not exposed to immune defences. HPVs are also well equipped to interfere with innate immune responses, and to delay adaptive immune responses [55]. In fact, one hypothesis is that the HPV oncogenes have evolved to evade the intrinsic immune system, and that it is these properties that inadvertently promote oncogenesis [28]. At some point during most HPV infections, the cell-mediated immune system is alerted to the infection and this induces regression of infected cells and lesions [55]. The role of the humoral immune response in natural infection is not clear; and many infected individuals do not seroconvert, leaving them vulnerable to subsequent infection by the same virus [54,55]. This is in contrast to the extremely high levels of humoral antibodies, and protection, that results from immunization with the HPV vaccine (see below) [56].

### (c) Latency

A debated question, and closely related topic, is whether HPVs enter a state of true latency (presence of viral DNA but no gene expression). It is challenging to detect low copies of HPV DNA in the persistently infected lower layers of an infected epithelium using *in situ* methods [57], never mind the rare stem-like cell that might harbour latent HPV. However, there is evidence from rabbit animal models that PV infection can become latent, with no apparent signs of infection [58,59]. Furthermore, latent infections can be reactivated by mechanical or cellular stress [59,60]. If these findings could be extrapolated to HPV infection, they would help explain the second wave of HPV infection that is often observed in older women [54]. These infections could be due to reinfection (by the same HPV type) in women who did not previously seroconvert in such a way as to gain protection, or could be due to reactivation of a latent infection.

### (d) Natural history of oropharyngeal infections

The link between HPV infection and head and neck cancer (HNSCC) was first noted over 30 years ago [61], and epidemiological evidence demonstrated a causal association of HPV in about 25% of these tumours [62]. HNSCC encompasses a wide range of tumours of the oral cavity, pharynx, larynx, nasal passages, sinuses and salivary glands, but as the prevalence of HPV association with each site has become better characterized, the association of HPV with oropharyngeal cancer (OPC) has risen to over 70% (figure 4*a*). This association may reflect the susceptibility of the epithelium in the tonsillar crypts to HPV infection and persistence [63]. Moreover, HPV-positive OPCs are becoming more prevalent than HPV-negative OPC tumours [64]. Men are particularly susceptible to acquiring HPV infection by oral sex, and slower to clear these acquired infections ([65,66] figure 4*c*). Increased vaginal exposure to HPV (as measured by number of sexual partners) is inversely proportional to the risk of oral HPV infection in women [65,66]. This has led to the hypothesis that women are more likely to develop a strong immune response resulting from genital HPV exposure, which protects them from subsequent oral infection [65,66].

### (e) Multiple infections

Many studies detect the presence of multiple HPV types at the same anatomical site within a single individual [35]. However, there is strong evidence that each infection is clonal and results from a single infected cell [67]. Even when multiple infections are detected at a single site, they are due to independent biological infections of adjacent tissue and as stated by Quint *et al*. ‘One virus, one lesion’ [67]. Moreover, epidemiological studies show that multiple infections do not synergize to increase the risk of oncogenesis [68].

### (f) Cells vulnerable to infection and oncogenesis

Different HPV types have tropism for different types, and anatomical sites, of cutaneous and mucosal epithelia. Although not well understood, this tropism is thought to be due to the transcriptional activity of each HPV type within permissive cells, rather than the requirement for specific cell surface receptors. All HPVs have a similar life cycle that requires establishment of infection within an epithelial basal cell, and generation of virus in the terminally differentiated progeny of the infected cell. However, the situation is more complex than this and the outcome of infection may depend on the precise nature of the originally infected cell. For example, most cases of cervical cancer arise from the cervical transformation zone, a region of the cervix where the ectocervix and endocervix meet and cells transition from squamous to columnar epithelial cells [69]. This site could be vulnerable to infection because the junction of two epithelial types increases accessibility of the proliferative basal cells. However, a discrete population of putatively residual embryonic, squamocolumnar junction cells have been identified and hypothesized to be the source of cells that give rise to HPV-associated tumours [69,70]. Notably, similar cellular transition zones exist in the oropharynx and anus, sites also highly susceptible to HPV oncogenesis, and similar cell populations have been identified in the anorectal junction [70]. Likewise, HPV oropharyngeal cancers typically arise from the highly specialized reticulated epithelium that lines the tonsillar crypts [64,71]. This specialized epithelium is in close contact with cells of the immune system, and is a frequent site of replication for several viruses.

The basal layers of stratified epithelia contain both slow-cycling stem-like cells, as well as proliferating transit-amplifying (TA) cells. The TA cells can divide both symmetrically (to generate more basal TA cells) and asymmetrically, where one of the daughter cells proceeds through the differentiation and tissue renewal process. Infection of a TA basal cell could result in a short-lived infection. In fact, modelling the stochastic dynamics of basal cells predicts that over 80% of infections could spontaneously clear as infected cells mature [72]. Long-term, persistent infection most probably requires infection of a slow-cycling stem cell. Moreover, infection of these slow-cycling cells could promote latency [73].

### (g) Genetic susceptibility to human papillomavirus infection

The importance of the immune system in controlling HPV infection is very evident in individuals with specific immunodeficiencies [74]. Individuals with EV, WHIM (warts, hypogammaglobulinemia, infections and myelokathexis) syndrome, GATA2 or DOCK8 deficiencies, and other syndromes are highly susceptible to pathological HPV infection by viral types that are often asymptomatic or self-limiting in normal individuals [74]. In some cases, these infections can progress to anogenital or skin cancer [74]. The latter observation has led the IARC to declare two beta-HPV types possibly carcinogenic (Group 2) in the genetic background of EV. Individuals with Fanconi anaemia (FA) have defects in DNA repair and are highly susceptible to HPV infections and carcinomas in sites usually associated with HPV oncogenesis. However, many of these cancers are HPV-negative; it seems that HPV activates the defective FA pathway, causing great genomic instability, and eventually rendering the cells no longer dependent on HPV to maintain the tumour phenotype [75].

### (h) Carcinogenic progression

Oncogenic E6 and E7 manipulate many cellular pathways to induce an environment that supports the viral life cycle, but inactivation of crucial cell-cycle checkpoints lead to genetic instability, accumulation of mutations in cellular genes and malignant progression. There are no mutations found consistently in all HPV-associated cancers, but there are frequent mutations in the PI3 K pathway, as well as in receptor tyrosine kinases, and genes related to keratinocyte differentiation and the immune response [76–78].

The viral genome in most, though not all, HPV-associated cancers is found integrated into the host genome. Integration can disrupt E2-mediated viral gene expression, thus promoting genomic instability by deregulating E6/E7 gene expression. E6/E7 are also expressed from integrated genomes as a viral-cellular fusion transcript that is often more stable than the viral mRNA [79]. There is also powerful selection for epigenetic events that promote E6/E7 expression [80,81]. Integration is thought to be an inadvertent event, but HPVs replicate adjacent to regions of the host DNA undergoing replication stress (fragile sites) and this could promote integration into these loci [82].

### (i) Multifactorial nature of human papillomavirus persistence and oncogenesis

Infection with an oncogenic HPV does not by itself place individuals at high risk of cancer, as most individuals clear infections within 1–2 years. However, long-term persistence of infection is key to the development of HPV-mediated cancer [83]. Persistence and oncogenesis are the cumulative result of many factors listed here: infected cells with stem-cell-like properties might be necessary to sustain a long-lived infection; the virus must establish a persistent infection in the face of intrinsic anti-viral factors; the HPV genome must be capable of robust long-term replication; the oncogenes of the virus need to inactivate cellular checkpoints; the virus must evade the cell-mediated immune system, which could recognize and clear the infection; stochastic genetic and epigenetic events can result in dysregulation of E6/E7 expression, and/or integration of the virus; and the infected cells might acquire properties that result in invasive cancer. On a positive note, many of these steps could be manipulated to intervene in the infectious process.

## 5. Therapeutics

HPV vaccines have been extremely successful from both a scientific and clinical viewpoint, and there is now a vaccine that protects against nine of the most prevalent HPVs associated with cancer and genital warts [56,84]. However, uptake has been slow in some countries, and the current vaccines are expensive and difficult to distribute to the developing world. For individuals already infected, the Pap smear test, introduced in 1941, has been very successful in screening for surgically treatable HPV-associated cervical lesions, but there is not an equivalent test for OPC because of the inaccessibility of the infection site. There are a number of potential therapeutic targets for HPV disease. Most infections are naturally cleared by cell-mediated immunity, and so therapeutic vaccines, or other immunomodulatory interventions have much potential [77]. Efficient partitioning of the viral genome is essential for persistent infection and disruption of this process could ‘cure’ genomes and resolve early lesions. HPV manipulates epigenetic modification of host chromatin [25] and integrated viral DNA is regulated by chromatin and DNA modifications [80,81], and so the rapidly expanding field of pharmacological modulation of epigenomes [85] could have great benefits for HPV-associated disease. Despite the accumulation of cellular mutations in HPV-associated cancers, the cells remain ‘addicted’ and dependent on continued expression of the viral oncogenes, providing another Achilles heel [44]. Strong support of these basic research areas will provide further insight into therapeutic interventions of HPV-associated disease.

## Data accessibility

This article has no additional data.
